# Metagenomic Insights into Anaerobic Metabolism along an Arctic Peat Soil Profile

**DOI:** 10.1371/journal.pone.0064659

**Published:** 2013-05-31

**Authors:** David A. Lipson, John Matthew Haggerty, Archana Srinivas, Theodore K. Raab, Shashank Sathe, Elizabeth A. Dinsdale

**Affiliations:** 1 San Diego State University, San Diego, California, United States of America; 2 Carnegie Institution for Science, Stanford, California, United States of America; National University of Singapore, Singapore

## Abstract

A metagenomic analysis was performed on a soil profile from a wet tundra site in northern Alaska. The goal was to link existing biogeochemical knowledge of the system with the organisms and genes responsible for the relevant metabolic pathways. We specifically investigated how the importance of iron (Fe) oxides and humic substances (HS) as terminal electron acceptors in this ecosystem is expressed genetically, and how respiratory and fermentative processes varied with soil depth into the active layer and into the upper permafrost. Overall, the metagenomes reflected a microbial community enriched in a diverse range of anaerobic pathways, with a preponderance of known Fe reducing species at all depths in the profile. The abundance of sequences associated with anaerobic metabolic processes generally increased with depth, while aerobic cytochrome c oxidases decreased. Methanogenesis genes and methanogen genomes followed the pattern of CH_4_ fluxes : they increased steeply with depth into the active layer, but declined somewhat over the transition zone between the lower active layer and the upper permafrost. The latter was relatively enriched in fermentative and anaerobic respiratory pathways. A survey of decaheme cytochromes (MtrA, MtrC and their homologs) revealed that this is a promising approach to identifying potential reducers of Fe(III) or HS, and indicated a possible role for Acidobacteria as Fe reducers in these soils. Methanogens appear to coexist in the same layers, though in lower abundance, with Fe reducing bacteria and other potential competitors, including acetogens. These observations provide a rich set of hypotheses for further targeted study.

## Introduction

Given the large carbon (C) pools in permafrost-affected soils and the rapid rates of climate warming at high latitudes [1], [2], an improved understanding of metabolic processes in Arctic soils would be valuable [3]. The advent of metagenomic sequencing has provided a powerful new tool for investigating the inner workings of microbial communities, including how their metabolic potential shapes biogeochemical cycles and how taxonomic and functional diversity are linked. At this time, only a small number of Arctic soil metagenomes have been published [4], [5], [6], representing very distinct environments (Canadian high Arctic, central Alaska black spruce forest and High Arctic fen in Svalbard). Our acidic wet tundra site in the Arctic coastal plain of northern Alaska contrasts with each of these sites, and may be unique in that anaerobic respiration using Fe(III) and/or humic substances (HS) as terminal electron acceptor contributes greatly to C cycling in this soil [7], [8], [9]. Because these electron acceptors are generally complex and insoluble, these processes occur through extracellular electron transport via outer membrane cytochromes [10]. Fe and HS respiration is widespread among prokaryotes [11], [12], but the genes involved in the majority of Fe-reducing species are not yet fully known [13], [14]. One major motivation for a metagenomic study of this soil was to see how the dominance of extracellular respiration manifests itself genetically. More generally, given the importance of the water table, oxygen concentration and redox state in controlling biogeochemistry in these soils [8], how does the relative abundance of respiratory and fermentative pathways change with depth in the active layer and into the upper level of the permafrost? The presence of Fe(III) and other alternative electron acceptors is generally inhibitory to methanogens [15] and the two processes appear to be negatively correlated at our site [8], but it is not known to what extent methanogens coexist spatially with Fe reducers in these soils or whether they are segregated by depth. Therefore, in this study we focus on anaerobic metabolism as revealed by metagenomic analysis of an Arctic peat soil profile that spans the active layer (0–30 cm in 10 cm increments) and the upper permafrost (30–40 cm).

## Materials and Methods

### Site Description

The study took place in a drained thaw lake basin in the Arctic coastal plain near Barrow, Alaska (“Biocomplexity Experiment,” 71.32°N, 156.62°W). Permission to use this site was provided by the Ukpeaġvik Iñupiat Corporation. The vegetation is dominated by mosses (*Sphagnum arcticum, S. tescorum, S. obtusum* and *S. orientale*) [16] and graminoids (*Carex aquatilis, Eriophorum scheuchzeri,* and *Dupontia fisheri*) [17]. In Feb 2006, frozen Cores were taken using a SIPRE corer at four random locations in the northern part of the basin. These were immediately stored at −40°C, and later sliced into 10 cm horizons with a power saw. Soil organic matter content (OM) was measured by loss on combustion. Soil pH was measured with a Thermo-Orion pH probe in saturated subsamples. Fe minerals were determined by extraction with acetate (for siderite) and citrate-dithionite (for reducible oxides) [18], followed by Fe determination by inductively coupled plasma spectroscopy. Anaerobic CO_2_ and CH_4_ production rates were measured by placing frozen soil samples in mason jars with lids fitted with septa, which were then flushed with N_2_ and incubated at 4°C. After soils thawed, the headspace was flushed to remove gases that were trapped in ice, and headspace was then sampled with a syringe at 3, 6 and 24 h, and analyzed by gas chromatography (SRI 8610C, Torrance CA, with Haysep column, FID and methanizer).

### DNA Extraction, Pyrosequencing and Metagenome Assembly

Frozen subsamples from each of the four cores were combined by horizon (∼5 g wet weight total) and DNA was extracted by alkaline lysis [19] after vortexing with glass beads for one minute, and precipitated with 30% PEG 6000/1.6 M NaCl at 4°C overnight. Approximately 500 ng of DNA was cleaned and processed according to protocol for the GS FLX Titanium Pyrosequencer [20]. DNA was randomly sequenced to provide a subset of all DNA found in the microbial community. DNA sequences were compared using the analysis platform MG-RAST version 3.2.2 [21]. Sequences underwent quality controls including the removal of tags and primers, sequences with redundant nucleotide series and dereplication [22]. All sequences were compared to known genes in the SEED database using BLASTX [23]. Sequence similarities to the database were refined to pairings with an e value of 10^−5^ and an alignment length of 50 base pairs. MG-RAST uses a subsystems approach to categorize DNA sequences relative to closest gene similarities [24].

### Data Analysis

Rarefaction analysis of annotated species richness was performed in MG-RAST. The curves plot the average number of distinct species annotations for subsamples of the complete dataset. Searches were performed in MG-RAST using SEED annotations, except in the case of decaheme cytochromes and PilA genes where additional annotations (GenBank, PATRIC) were used and redundant sequences were deleted. To search for decaheme cytochrome genes that were not annotated in the SEED, we performed TBLASTN (e-value <10^−5^) against the metagenomes using protein sequences from NCBI. The relative abundance of sequences was represented as a percentage of total sequences in each metagenome. The Pearson chi-squared statistic (χ^2^) was calculated to compare the expected *vs.* observed proportion of sequences among soil layers or other categories, testing the null hypothesis that genes were distributed evenly. Results are defined to be significant at P<0.05 and marginally significant at 0.05≤P<0.1. Protein sequences of decaheme cytochromes were initially aligned using the ClustalW program in BioEdit [25], and then manually adjusted using the ten heme-binding motifs (CxxCH) and other conserved features. A protein maximum likelihood tree was generated using ProML. The Waseca Farm Soil metagenome, available at MG-RAST (http://metagenomics.anl.gov/), was used for comparative purposes. This was a surface soil (0–10 cm) from a farm in Waseca County, Minnesota, described as a clay loam, with fair to low OM content [26]. The Waseca metagenome is similar in size (138,347 sequences) to those in our study. The Barrow soil metagenomes were submitted to the GenBank Sequence Read Archives (www.ncbi.nlm.nih.gov/Traces/sra/) and assigned the accession number SRP020650.

## Results and Discussion

### Characteristics of Soils and Metagenomes

The mean OM content in the soils shows the two upper soil layers (0–10 and 10–20 cm) resided mainly in the organic horizon and the deeper two layers, the mineral horizon (Table 1). The active layer depth at this site has been measured to be ∼30 cm [27], and so the 30–40 cm layer may be considered to be upper permafrost (though it is possible that the upper parts of these samples have occasionally thawed in recent history). The mean pH of all soil horizons was mildly acidic, as is typical for this site; the pH of the surface horizon varies spatially and seasonally as redox conditions change and protons are consumed or released by Fe reduction or oxidation, with an overall mean (±SD) pH of 4.8±0.6 [8]. The relatively high pH in the surface layer at the time of measurement in the laboratory indicates these soils were in a reduced state. Extractable Fe minerals were generally higher in the mineral horizon, but both substrates (reducible Fe oxides and hydroxides such as goethite and ferrihydrite) and products (siderite) of Fe reduction were abundant at all depths (Table 1). CO_2_ and CH_4_ fluxes in anaerobic incubations were variable among replicates but trended towards highest respiration rates at 0–10 cm and peak methanogenesis rates at 20–30 cm (Table 1). The ratios of CO_2_:CH_4_ production in these incubations were quite high (back-transformed geometric means range from 37 to 916), consistent with published observations from this site [8], [17], [28], reflecting high availability of alternative electron acceptors such as Fe(III) in these soils [15], [29], [30], [31].

**Table 1:** Characteristics of soils used in metagenomic analysis.

Layer (cm)	OM^a^ (%)	pH	Ac-Fe^b^ (mg cm^−3^)	CD-Fe^c^ (mg cm^−3^)	CO_2_ (nmolecm^−3 ^h^−1^)	CH_4_ (nmole cm^−3 ^h^−1^)
0–10	87.2 (2.0)	6.36	0.498 (0.310)	0.402 (0.130)	9.67 (3.80)	0.012 (0.005)
10–20	90.0 (0.3)	4.63	0.136 (0.020)	0.254 (0.006)	2.72 (0.64)	0.009 (0.003)
20–30	39.3 (5.5)	4.46 (0.04)	1.289 (0.831)	0.858 (0.256)	3.14 (1.06)	0.254 (0.161)
30–40	26.3 (5.0)	5.45 (0.16)	0.762	0.854 (0.133)	4.81 (2.97)	0.026 (0.023)

a - Organic matter = OM.

b -Ac-Fe = sodium acetate-extractable Fe, “siderite”.

c -CD-Fe = citrate/dithionite-extractable Fe, “reducible Fe oxides”.

Legend: Values are means (and standard errors, where available). Rates of CO_2_ and CH_4_ production are from anaerobic incubations at 4°C.

**Figure 1:**
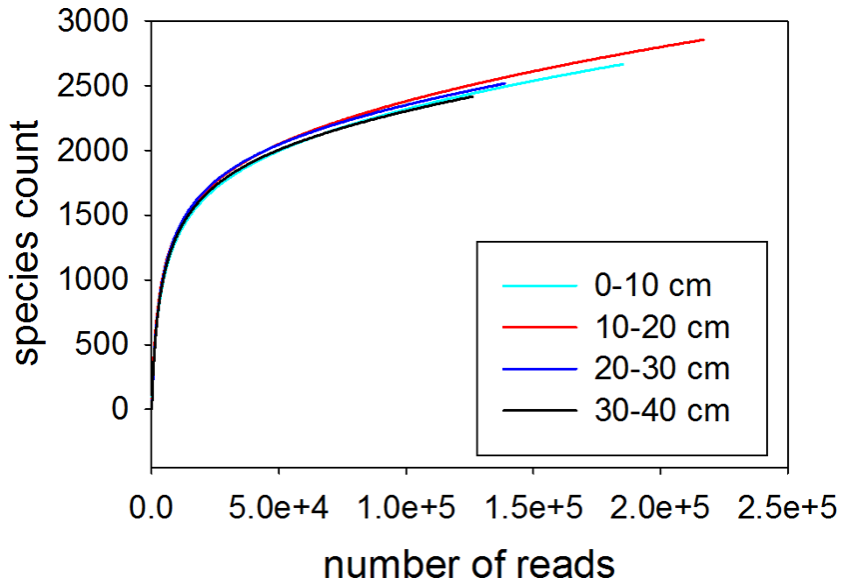
Rarefaction curve of annotated species richness as a function of the number of sequences sampled.

The metagenomic libraries produced from the upper two soil layers produced a higher yield of sequences than the lower two layers, though the sequence quality was comparable across depths (Table 2). After quality control, 54.4% of all sequences were categorized as known proteins. Rarefaction analysis showed that decent coverage was achieved in all four libraries in terms of annotated species diversity, in that the slopes of the curves decline markedly with increasing sequences (Figure 1).

**Table 2:** Characteristics of metagenomes.

Layer	0–10 cm	10–20 cm	20–30 cm	30–40 cm
Pre-QC sequences	185,235	216,879	138,794	126,219
failed QC	56,865	57,809	48,030	47,123
Post-QC sequences	128,370	159,070	90,764	79,096
known proteins	71,640	88,712	44,269	44,190
unknown proteins	55,850	68,547	40,959	34,071
rRNA	688	900	5,536	534
other	192	911	0	301
Post-QC length (bp)	450±90	459±84	429±118	448±96
G+C (%)	57±9	53±11	53±10	52±11

### Distribution of Functional Genes

**Figure 2:**
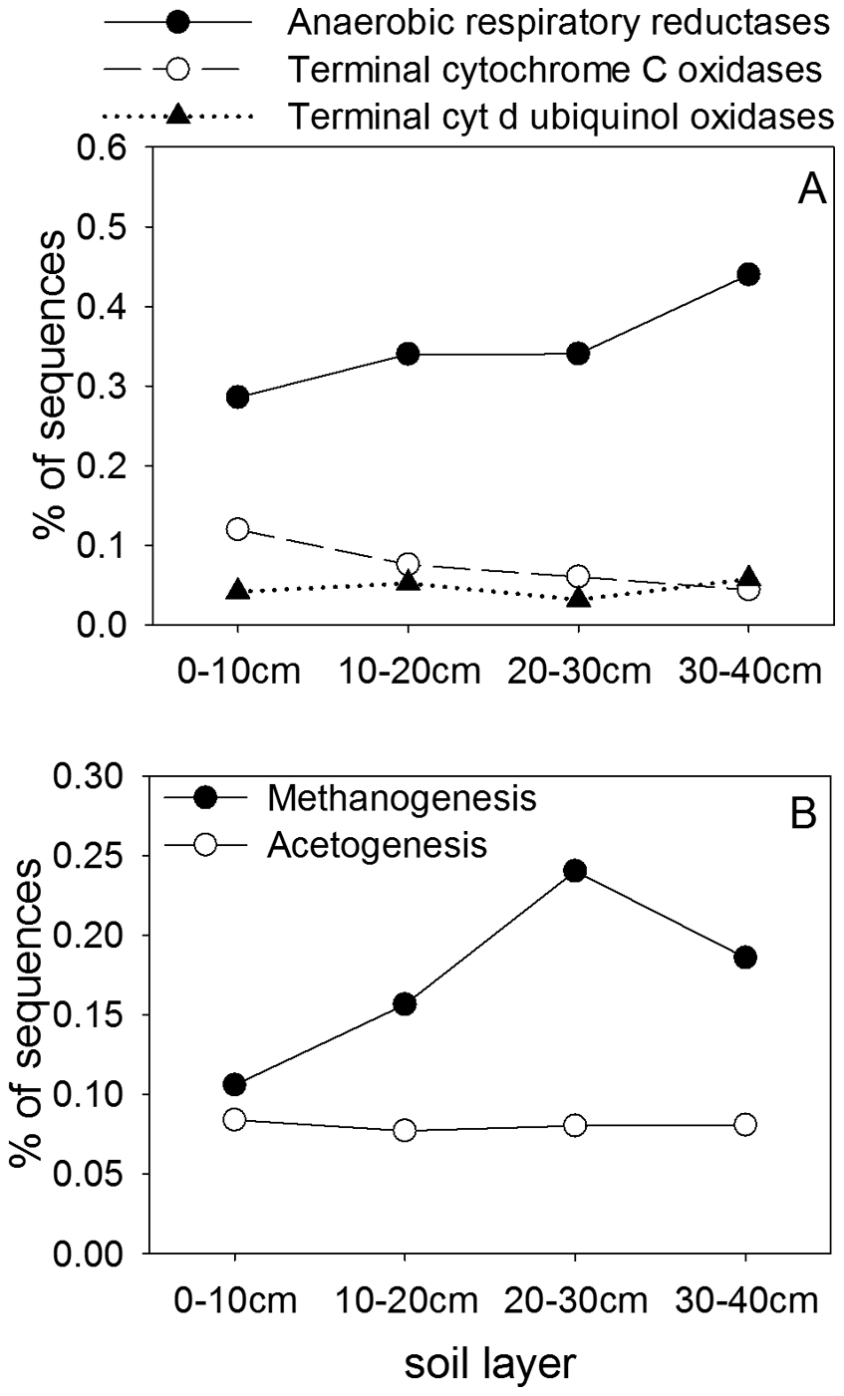
Percent abundance of sequences in the metagenomes with similarities to genes from various respiratory pathways. Anaerobic respiratory reductases include the SEED subsystem of the same name plus other anaerobic respiration-related genes (denitrification, sulfate reduction, reductive dechlorination, tetrathionate respiration, TMAO reduction and decaheme cytochromes). Methanogenesis genes include all related functions in SEED, including methanopterin biosynthesis. Acetogenesis genes are CO dehydrogenases and related functions. χ^2^ results were highly significant (P<0.001) for anaerobic reductases (χ^2^ = 33.14), cytochrome C oxidases (χ^ 2^ = 41.99) and methanogenesis (χ^ 2^ = 61.28), significant for cytochrome d oxidases (χ^ 2^ = 8.16, P = 0.043) and non-significant for acetogenesis (χ^2^ = 0.411, P = 0.938).

The relative number of sequences classified by SEED as anaerobic respiratory reductases increased with depth, while terminal cytochrome C sequences (involved in aerobic respiration) declined with depth (Figure 2A). Terminal cytochrome d ubiquinol oxidases, which are active at low levels of O_2_ [32], showed no clear trend with depth and the variations among layers were barely significant. Methanogenesis genes followed the same trend as CH_4_ fluxes in laboratory incubations, being lowest in the shallowest layer and peaking at 20–30 cm (Figure 2B). Acetogenesis genes were less abundant compared to methanogenesis genes, and did not change significantly with depth. As methanogenesis is an obligately anaerobic process, the increase in genes with depth through the active layer is easily explained by decreasing O_2_ and redox levels. The decline in the upper permafrost layer (30–40 cm) could be explained by nearly constant subzero temperatures, as methanogenesis is thermodynamically marginal and highly temperature sensitive [33], [34].

A wide variety of anaerobic respiration pathways were represented, indicating the potential use as terminal electron acceptors of nitrate, sulfate, arsenate, Fe(III)/HS, dimethylsulfoxide (DMSO), trimethylamine N-oxide (TMAO) and organic chlorine compounds (Table 3). Decaheme cyctochromes are essential for extracellular respiration of Fe(III) in *Shewanella* and are found in the genomes of many other Fe reducing species [35], [36], [37], [38], [39]. Therefore we used these genes as indicators of Fe/HS reduction (see further discussion below). The relative abundance of the pathways in Table 3 depends not only on the importance of the pathway in these soils, but also on the complexity and annotation of the pathways. Based on other molecular and biogeochemical evidence, Fe reduction is the dominant anaerobic pathway in these soils [7], [8]. The decaheme cytochromes are fairly abundant relative to the other pathways in Table 3 considering that they represent only two genes (MtrA and MtrC) among many required for Fe reduction [40], [41], in a subset of all Fe- and HS-reducing microbes. Denitrification and sulfate reduction are better understood and include a variety of genes in the annotation. However, decaheme cytochromes are also involved in other extracellular electron transport processes, such as DMSO respiration and Fe(II) oxidation [14], [39], [42], [43]. The large number of sequences with similarities to arsenate reductases seems surprising given that As was present in a soil profile from a similar, medium-aged DTLB at 13±1 µg g^−1^, more than 1000 fold lower than Fe in these soils (Raab, unpublished data). However, these genes may serve primarily in detoxification rather than energy generation. In fact the majority of these sequences most closely matched arsenate reductase glutaredoxin (E.C. 1.20.4.1), which is not involved in energy generation.

**Table 3:** Relative abundance (%) and total number of sequences matching genes from various anaerobic respiratory pathways.

Layer	Denitrifi-cation	Sulfatereduction	Arsenate reduction	10-heme cyto^a^	DMSO^b^ reduction	TMAO^c^reduction	Dehalo-resp^d^
0–10 cm (%)	0.027	0.012	0.016	0.026	0.009	0.007	0.002
10–20 cm (%)	0.030	0.029	0.019	0.019	0.008	0.008	0.001
20–30 cm (%)	0.017	0.014	0.026	0.014	0.012	0.014	0.004
30–40 cm (%)	0.046	0.023	0.015	0.010	0.018	0.009	0.000
Mean (se) (%)	0.030 (0.006)	0.019 (0.004)	0.019 (0.003)	0.018 (0.003)	0.012 (0.002)	0.010 (0.002)	0.002 (0.001)
total seqs	134	92	86	84	49	42	7
Pearson χ^2^ (P)	12.26 (0.007)	12.42 (0.006)	4.15 (0.246)	6.92 (0.074)	4.14 (0.247)	6.36 (0.096)	3.50 (0.321)

a -10-heme cyto = decaheme cytochromes.

b -DMSO = dimethylsulfoxide.

c -TMAO = trimethylamine N-oxide.

d -Dehaloresp = dehalorespiration.

**Figure 3:**
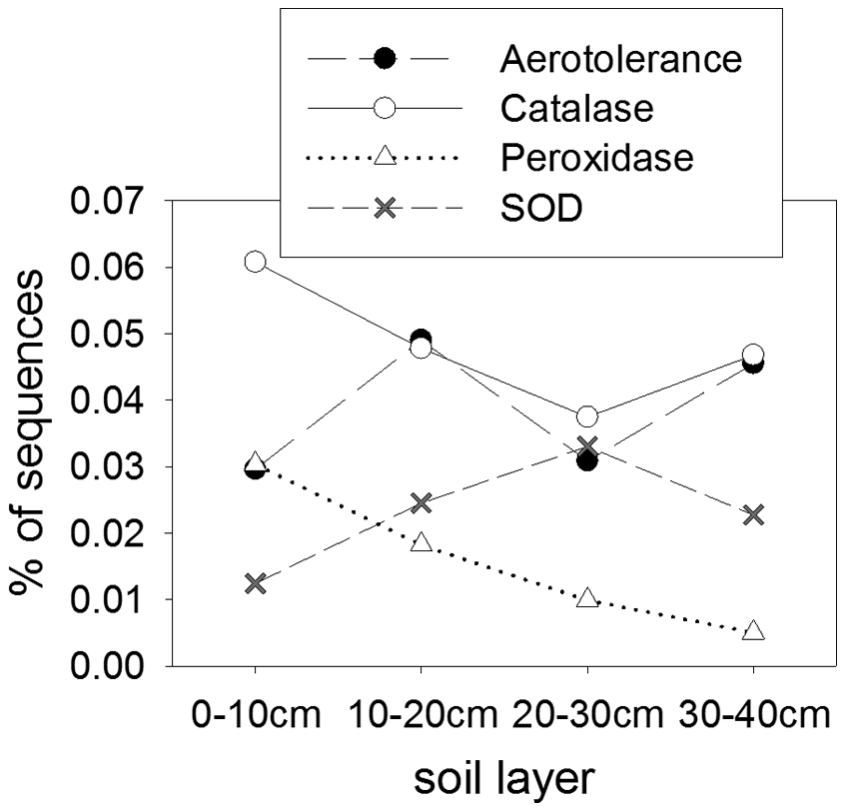
Percent abundance of sequences in the metagenomes with similarities to oxidative stress genes. Aerotolerance refers to the SEED subsystem known as “Aerotolerance operon in Bacteroides and potentially orthologous operons in other organisms.” χ^2^ results were highly significant for peroxidase (χ^2^ = 21.37, P<0.001), significant for aerotolerance (χ^2^ = 10.03, P<0.018) and SOD (χ^2^ = 11.08, P = 0.0113), and marginally significant for catalase (χ^2^ = 6.41, P = 0.093).

Genes for coping with oxidative stress were found at all layers, though the trends in abundance were complex and somewhat hard to interpret (Figure 3). Taken collectively, the abundance of all genes in the oxidative stress subsystem of SEED did not change with depth (data not shown, mean relative abundance for all depths = 0.564%, χ^2^ = 2.62, P = 0.454). Peroxidase genes, important in facultative anaerobes [44], declined with depth, consistent with a community shift from facultative to obligate anaerobes with depth. Catalase and superoxide dismutase (SOD) showed inverse patterns, possibly reflecting an either/or strategy for these two genes. While these genes are required by aerobes, many strict anaerobes are known to possess [44], [45] and express them in response to O_2_ exposure [46]. The SOD genes found in the lowest layer matched those from genomes of strict anaerobes such as sulfate reducers, syntrophic bacteria and methanogens, though facultative genera (such as *Rhodopseudomonas*) and strict aerobes (such as *Flavobacterium*) were also represented. In this ecosystem the water table occasionally drops well below the surface, especially in hot, dry years [17]. Additionally, O_2_ could be transported to depth by aerenchymous graminoid roots [47]. It would therefore serve these microbial communities to tolerate occasional inputs of O_2_.

**Figure 4:**
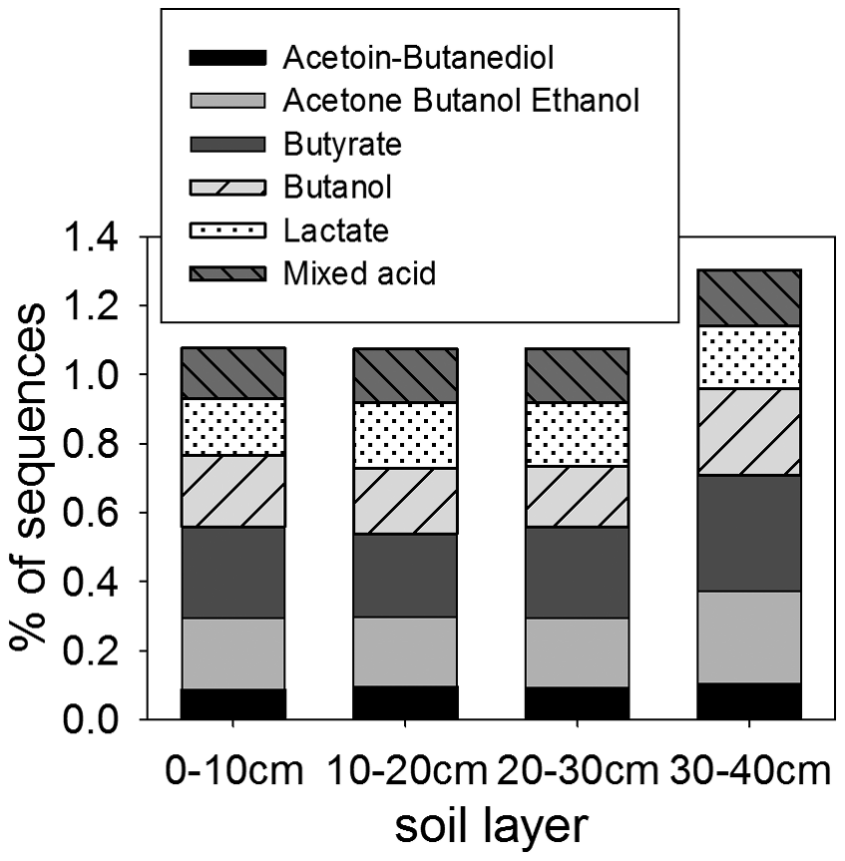
Percent abundance of sequences with similarities to genes within SEED fermentation subsystems (χ^2^ = 30.88, P<0.001).

Genes within the SEED Fermentation subsystems were abundant at all depths, especially in the upper permafrost (Figure 4). The distribution of these subsystems stayed fairly constant, with butanol-related genes dominating: the three most abundant fermentation genes were acetyl-CoA acetyl transferase (E.C. 2.3.1.9), butyryl-CoA dehydrogenase (1.3.99.2) and enoyl-CoA hydratase (or crotonase, 4.2.1.17), all found in the butanol pathway [48].

### Distribution of Taxa Associated with Known Functions

**Figure 5:**
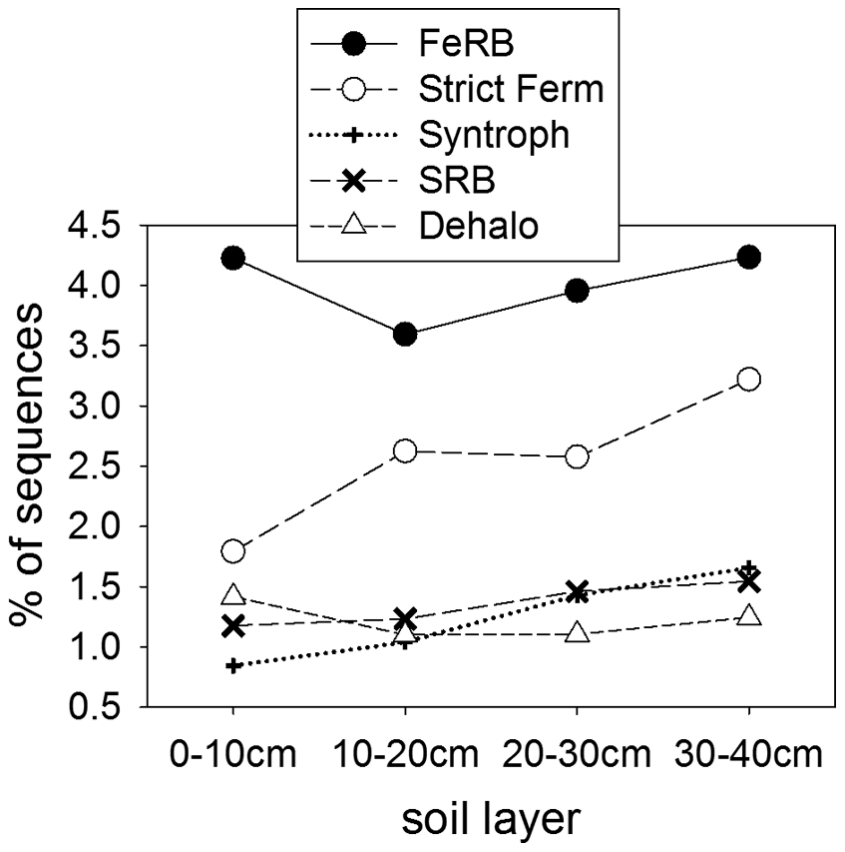
Percent abundance of genomic sequences from selected taxa with known respiratory pathways. FeRB, Fe-reducing bacteria consist of Geobacteraceae (incuding *Pelobacter* and *Desulfomonas*), *Rhodoferax (Albidiferax) ferrireducens, Shewanella, Carboxydothermus* and *Anaeromyxobacter*. SRB, sulfate reducing bacteria include the Desulfobacterales, Desulfovibrionales and Desulfurococcales. Dehalo, Dehalorespirers include *Anaeromyxobacter*, *Carboxydothermus, Dechloromonas*, and *Dehalococcoides*. Strict fermenters (Strict Ferm) include Clostridiales and *Bacteroides*. Syntrophic bacteria include Syntrophaceae, Syntrophobacteraceae and Syntrophomonadaceae. All the taxa shown varied significantly with depth (P<0.001) by the Pearson chi-square test.

Sequences matching known Fe-reducing bacterial species were more abundant than those associated with other anaerobic pathways (Figure 5). The slight drop in Fe-reducing bacteria between 0–10 and 10–20 cm could relate to depletion of Fe in this layer (see Table 1). The syntrophic bacteria, sulfate-reducing bacteria and the predominantly strict fermenters, *Clostridia* and *Bacteroides,* all increased with depth as would be expected for strictly anaerobic species. Sequences related to dehalorespiring taxa were present in comparable levels to sulfate-reducing bacteria, but were highest near the surface. The ability to use organic chlorine (Cl) compounds as electron acceptors is mainly considered in relation to contamination from perchloroethylene and other solvents [49]. However, naturally-occurring organic Cl compounds occur in relatively pristine ecosystems such as forest soils [50]. The surface layer might be richest in organic Cl compounds, having the highest OM content, being closest to inputs of Cl^−^ from rain, and being most subject to oxidative reactions that can lead to the production of reactive Cl compounds and chlorination of OM [51]. On the other hand, it has been suggested that organic Cl compounds can form abiotically as a result of Fe(III) reduction [52], a mechanism likely to occur in anoxic layers of these soils. Despite the growing recognition that Cl cycling in soils is dynamic and biologically-driven [53], the role of naturally-occurring organic Cl compounds as electron acceptors in pristine habitats has received little attention [54].

**Figure 6:**
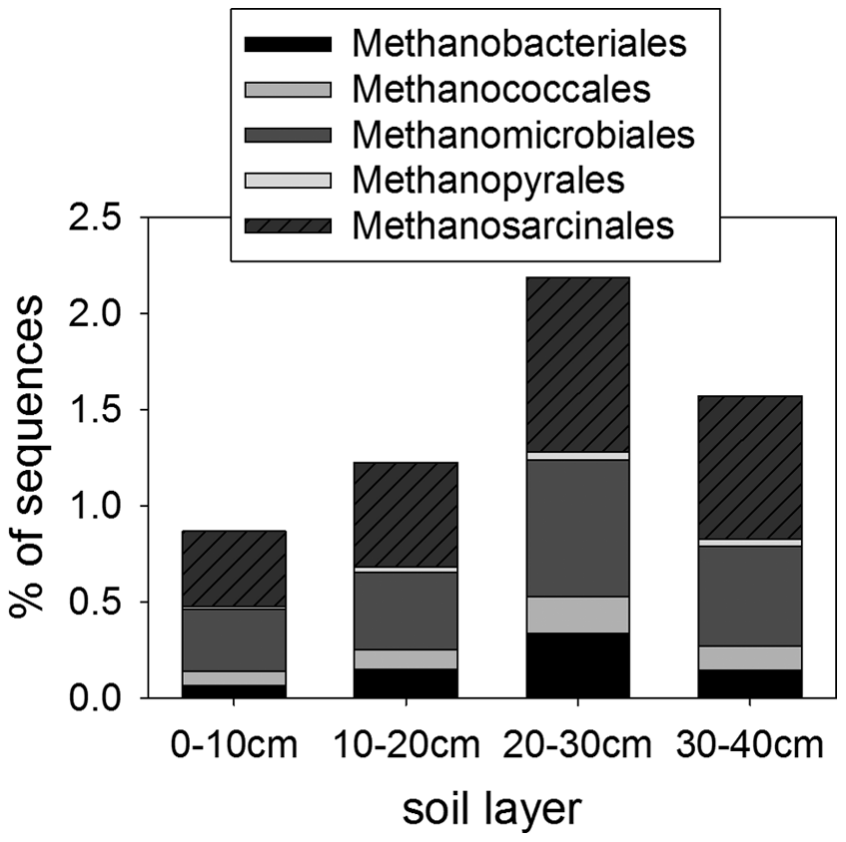
Percent abundance of sequences from methanogenic Archaea genomes with depth (χ^2^ = 61.29, P<0.001).

The relative abundance of sequences from methanogen genomes followed the same pattern as CH_4_ flux and methanogenesis genes, peaking in the 20–30 cm layer (Figure 6). That methanogenic DNA sequences make up only 1.4% of the community (with the rest predominantly bacterial sequences) is consistent with the high CO_2_:CH_4_ ratios in this ecosystem and the high levels of Fe(III) and other alternative electron acceptors, as discussed earlier. The most abundant order of methanogens was the Methanosarcinales (44% overall), the only group capable of producing CH_4_ from acetate as well as the more widespread H_2_/CO_2_ pathway [55]. It is estimated that the acetoclastic pathway accounts for about two thirds of CH_4_ from most ecosystems [56] including subarctic peat [34], and so the predominance of the Methanosarcinales among methanogens is not surprising.

### Comparison to a Reference Soil Metagenome

To provide perspective on the relative abundances of the functional genes and phylogenetic groups presented above, we performed the same analysis on a metagenome of an agricultural surface soil (Waseca farm soil) (Table 4). The farm soil had a significantly higher abundance of terminal cytochrome C oxidases and lower levels of terminal cytochrome d ubiquinol oxidases, indicating a higher prevalence of aerobic metabolism and lower affinity for microaerobic conditions. Methanogenesis and sulfate reduction pathways were more enriched in the Barrow soil, as were genomes of methanogens and sulfate reducing bacteria, while denitrification genes were more abundant in the farm soil. Both methanogenesis and sulfate reduction are carried out by strict anaerobes and occur at lower redox potentials than denitrification, generally a facultative process, and so these results indicate the more anoxic nature of the Barrow soil. Similarly, genomes of syntrophic bacteria and the strict fermenters, Clostridiales and *Bacteroides*, were more abundant in the Barrow soil (especially at depth, Figure 5). However, the farm soil metagenome contained comparable amounts of several anaerobic pathways, including Fe reducing bacteria, and higher levels of some categories, such as dehalorespiring bacteria. The Waseca farm soil metagenome also had a similar abundance of anaerobic respiratory reductases to the overall value in the Barrow profile (though less than the deeper layers). The farm soil was a clay loam, and anaerobic microsites are common in fine textured soils [57]. Overall, the comparison confirms the anaerobic nature of the Barrow soil metagenome, but shows that anaerobic pathways can also be common in well-drained surface soils. These metagenomes represent the potential metabolism of the microbial community, while gene expression profiles probably differ more drastically between the farm soil and the arctic peat.

**Table 4:** Comparison of Barrow soil metagenomes presented in this study (all layers combined) with published Waseca farm soil metagenome [6].

	Waseca (%)	Barrow (%)	Pearson^a^ χ^2^	P^a^
**Functional genes**				
Anaerobic reductases	0.248	0.266	1.299	0.2545
Terminal cytochrome C oxidases	0.168	0.080	82.819	0.0001
Terminal cytochrome d ubiquinol oxidases	0.023	0.047	14.289	0.0002
Methanogenesis	0.035	0.164	131.527	0.0001
Acetogenesis	0.080	0.080	0	1
Fermentation	1.239	1.115	14.346	0.0002
Decaheme cytochromes	0.009	0.018	5.897	0.0152
Denitrification	0.095	0.029	103.052	0.0001
Sulfate reduction	0.009	0.020	6.535	0.0106
TMAO reduction	0.012	0.009	0.892	0.3449
DMSO reduction	0.016	0.011	2.905	0.0883
As reduction	0.025	0.019	2.277	0.1313
Dehalorespiration	0.000	0.002	2.8	0.0943
Oxidative stress	0.770	0.564	73.088	0.0001
Catalase	0.041	0.049	1.28	0.258
SOD	0.020	0.023	0.173	0.6775
Peroxidase	0.020	0.018	0.467	0.4943
Aerotolerance (Bacteroides)	0.048	0.039	1.85	0.1738
**Genomes**				
Methanogens	0.765	1.377	322.21	0.0001
Fe reducing bacteria	4.070	3.954	3.578	0.0586
Dehalorespirers	2.232	1.214	758.443	0.0001
Sulfate reducing bacteria	0.860	1.316	182.283	0.0001
Syntrophic bacteria	0.502	1.168	464.345	0.0001
Strict Fermenters (Clostridiales and Bacteroides)	2.350	2.484	7.848	0.0051

a Pearson chi-squared statistic (χ^2^, and corresponding P value) compares proportions of sequences between the two soils.

### Taxonomic Assignments of Key Functional Genes

The community of potential fermenters was diverse, with pathways for ethanol, butanol and lactate fermentation found in many phyla (Table 5). One striking trend was that Bacteroidetes sequences were not well represented among the alcohol dehydrogenase (ADH) or butanol genes but dominated lactate dehydrogenase (LDH) genes. The reverse pattern was true for Actinobacteria and Firmicutes, while Acidobacteria in these soils seemed to specialize in ethanol fermentation. The Proteobacteria were well represented in all three pathways.

**Table 5:** Taxonomic representation of key genes in fermentative pathways found in all four metagenomes.

		Alcoholdehydrogenase (%)	Butanolgenes^a^ (%)	Lactatedehydrogenase (%)	Pearson χ^2^(P)^c^
Acidobacteria		12	5	4	22.25 (<0.001)
Actinobacteria		17	13	4	24.79 (<0.001)
Bacteroidetes		4	5	35	214.1 (<0.001)
Firmicutes		12	16	4	23.15 (<0.001)
Proteobacteria	α	16	19	16	1.58 (0.453)
	β	16	15	9	6.97 (0.031)
	γ	10	8	8	1.36 (0.507)
	δ	9	11	9	1.35 (0.510)
Other phyla^b^		4	8	10	11.37 (0.003)

a includes butyryl and 3-hydroxybutyryl-CoA hydrogenases, enoyl-CoA hydratase, and acetyl-CoA acetyltransferase.

b includes (in decreasing order) Chloroflexi, Euryarchaeota, Verrucomicrobia, Cyanobacteria, Deinococcus, Planctomycetes and Spirochaetes.

c tests the null hypothesis that the percentages are the same across the three columns.

Legend: Similarities to these genes (as annotated in SEED) were combined by the genus in which they were found and the 50 most abundant genera for each gene were aggregated into phyla (or classes for the Proteobacteria).

**Figure 7:**
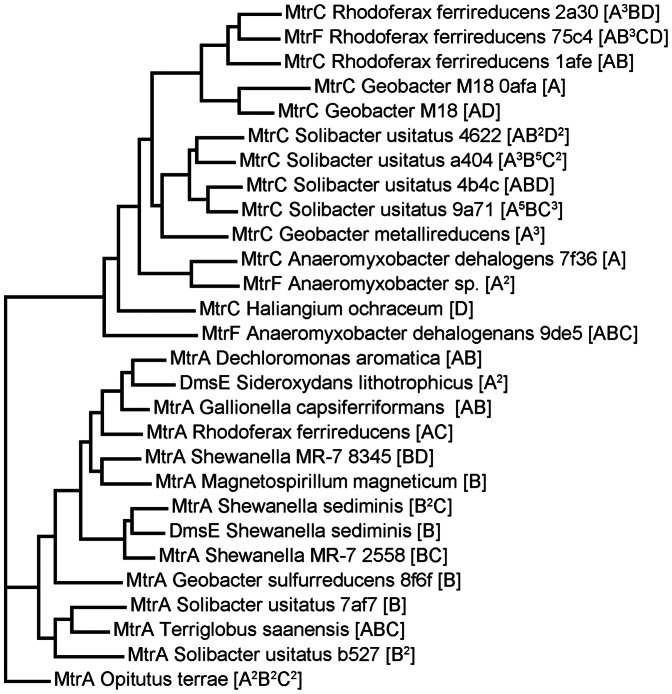
Maximum likelihood phylogenetic tree of decaheme cytochrome protein sequences with high similarity to metagenomic sequences. (Mean log E value = −13.3, mean % identity = 70.5%, mean alignment length = 47.7). The code in square brackets represents the number of similar sequences (superscript) from the four layers, with A-D corresponding to shallow-deep. Different sequences from the same genome are differentiated by the last 4 digits of the MD5 number in the M5nr database (http://tools.metagenomics.anl.gov/m5nr/m5nr.cgi). Sequences were aligned based on the ten CxxCH heme-binding domains. MtrA Opitutus has only 7–8 canonical heme-binding domains (eighth domain = TxxCH), and so this sequence was used as an outgroup.

The ability to perform dissimilatory Fe reduction is widespread throughout the microbial world, and there does not appear to be a single, universal genetic pathway for this process [11]. The genes involved in Fe reduction are best described for *Shewanella oneidensis* MR-1, and while homologs of these genes are not found in all Fe reducers, a common theme is the importance of multiheme cytochromes such as CymA, MtrA, MtrC and OmcA in *Shewanella*, [13] and OmcE, OmcS and OmcZ in *Geobacter sulfurreducens* [58], [59]. To shed light onto the potential diversity of Fe reducing bacteria beyond the best studied genera, we compiled the decaheme cytochromes that were most closely matched by metagenomic sequences in this study (Figure 7). (These do not include the OmcESZ genes from *Geobacter sulfurreducens*, which are tetraheme and octaheme cytochromes [58], however these genes were not found in either the MG-RAST annotation nor in TBLASTX searches of the metagenomes). These sequences fell into two main groups: genes annotated as MtrA or DmsE and those annotated as MtrC, MtrF or OmcA. In *Shewanella*, MtrA (embedded in the inner leaflet of the outer membrane) transfers electrons via the porin, MtrB, to decaheme cytochromes on the cell surface, MtrC and OmcA, the terminal reductases for Fe(III) oxides [13]. MtrA may also be capable of reducing chelated forms of Fe [37]. DmsE is a homolog of MtrA in *Shewanella* used in extracellular DMSO respiration [14]. Figure 7 includes many known genera capable of Fe(III) reduction (*Shewanella, Geobacter, Magnetospirillum, Rhodoferax, Anaeromyxobacter*). In the current literature there is no mention of MtrC homologs in *Geobacter* species, but recently published genomes for *Geobacter metallireducens* and *Geobacter* strain M18 include annotations for MtrC. Furthermore, MtrA homologs have been noted in *Geobacter* [14], and their potential role in extracellular respiration has been postulated [37]. The role of decaheme cytochromes in Fe reduction in *Rhodoferax ferrireducens* is not yet known, but the genome contains several which may be of importance [35]. Decaheme cytochromes are widespread among Gram negative bacteria [38], and it has recently been shown that Fe(III) reduction in Gram positive bacteria may involve decaheme and other multiheme cytochromes [36]. It is likely that many of these decaheme cytochromes play a role in outer membrane electron transport. In fact, most of the MtrC genes in Figure 7 include putative hematite-binding motifs (S/T-P-S/T), and two (*Geobacter* sp. M18 and *Rhodoferax*) contain the conserved motif of hematite-binding peptides generated in an *in situ* evolution experiment (S/T-x-S/T-P-S/T) [60]. The presence of this motif provides further evidence that these genes may be involved in Fe oxide reduction in organisms other than *Shewanella*.

Interestingly, numerous Acidobacterial sequences (*Solibacter usitatus* and *Terriglobus saanensis*) were also found in this analysis (Figure 7). Acidobacteria dominate soils but their physiology is still mysterious, being underrepresented in pure culture [61], [62]. The phylum includes at least one known Fe-reducing species, *Geothrix fermentans* [63]. Given the importance of Fe reduction in this ecosystem, the abundance of Acidobacteria in this soil and the relatedness of Acidobacterial decaheme cytochromes to those from known Fe reducers (e.g. *Geobacter metallireducens,* Figure 7), it is likely that Acidobacteria contribute to Fe reduction in this ecosystem.

Fe(II) oxidizing species were represented among a cluster of related MtrA genes, including the microaerophilic species, *Gallionella* and *Sideroxydans,* and the nitrate-dependent Fe(II) oxidizer, *Dechloromonas* [11] (Figure 7). MtrA homologs (PioA) are required for Fe oxidation in some species, possibly indicating that electrons can flow both ways through these multiheme-metal systems [43]. The search for similarities to decaheme cytochromes in the metagenomes identified cytochromes with varying numbers of heme-binding motifs, including 7 hemes from *Carboxydothermus hydrogenoformans*, 8 from *Chthonibacter flavus*, 9 from *Anaeromyxobacter* spp., 15 from *Geobacter sulfurreducens*, 16 from *Koribacter versatilis*, 19 from *Paludibacter propiocigenes*, 20 from *Maribacter* sp., and 22 from *Leptothrix cholodnii*.

**Figure 8:**
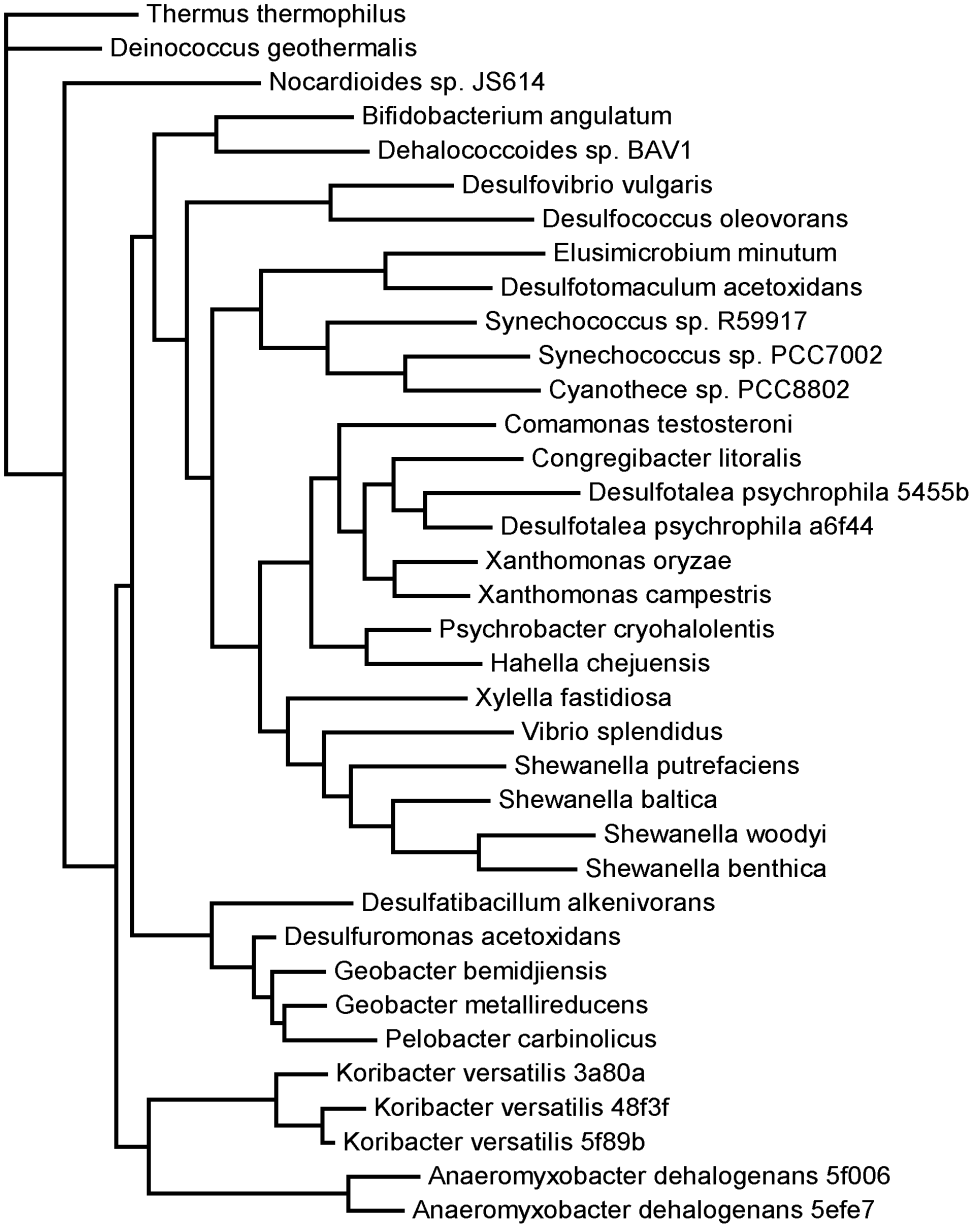
Maximum likelihood tree of PilA genes with highest similarity to metagenomic sequences. Sequences from the same genome are differentiated by the last 5 digits of the MD5 number in the M5nr database (http://tools.metagenomics.anl.gov/m5nr/m5nr.cgi).

The pilus protein, PilA, forms conductive nanowires in *Geobacter* biofilms that allow them to reduce Fe oxides or electrodes of bioelectrochemical systems [64]. Similar conductive structures have been found in *Shewanella* and the Cyanobacterium, *Synechocystis*, but *Geobacter* appears to be unique in not requiring outer membrane cytochromes for this conductivity [65], [66]. The metagenomes contained sequences that matched PilA genes from diverse bacterial taxa (Figure 8). Numerous Fe reducers were represented, including species within the *Geobacteraceae*, as well as *Anaeromyxobacter* and *Shewanella* species. As observed by others [64], PilA genes in the Geobacteraceae are shorter than those in other species, possibly contributing to their conductivity [65]. Three sequences from the Acidobacterium, *Candiatus Koribacter usitatus*, were found, suggesting the possibility that Acidobacterial PilA genes may contribute to conductive biofilms in these soils.

### Conclusions

These data demonstrate that the soil microbial community in the Barrow soil ecosystem is predominately geared toward anaerobic metabolism. A diverse range of respiratory and fermentative pathways are represented and diverse taxonomic groups partake in these pathways. Surveying decaheme cytochromes appeared to be a useful approach for studying the potential diversity of Fe-reducing bacteria, and indicated a likely role for Acidobacteria in Fe reduction in these soils. In terms of changes in gene abundance through the profile, anaerobic pathways predictably tended to increase with depth. Despite this, the upper permafrost (30–40 cm) was qualitatively similar to the active layer (0–30 cm), though relatively enriched in anaerobic respiration and fermentation pathways while having fewer methanogenesis genes than the lower part of the active layer (20–30 cm). This result contrasts with those of a previous metagenomic study of Arctic soil [4], which found a higher overall abundance of methanogens compared to the current study, and comparable or higher levels in the permafrost than in the active layer. The chemistry of those soils (from a black spruce forest at a lower latitude with discontinuous permafrost) was different from the *Sphagnum*-dominated peat studied here, having a much deeper organic layer and higher pH. In particular the thinner organic layer, and hence the more accessible mineral sub-layer, of the Barrow soil is probably responsible for increased Fe(III) availability, in turn diminishing methanogenesis. The proportion of genomic sequences from methanogens in our study was comparable to that found in two high Arctic fens in Svalbard [6], which are similar to the Barrow, Alaska site in that they are mildly acidic, moss-dominated high Arctic soils, although they have different dominant plant species and a thicker organic layer. In contrast to the study presented here, in the deep layers of the Svalbard soils fermentation genes dominated over anaerobic respiratory pathways [6]. Again, this may have resulted from the more accessible mineral layer providing Fe(III) as an alternative electron acceptor in the Barrow soils.

One question we asked was how methanogens coexisted with Fe-reducers in this soil given the high amount of available Fe(III) and the thermodynamic advantage to this pathway. One possibility was that methanogens were restricted to a deeper layer where Fe reduction was less prevalent; however both methanogens and Fe-reducers were found in all layers, suggesting the two processes can coexist (at least spatially if not temporally). One explanation could be reduced competition pressure due to relatively high fluxes of energy through this organic rich system [67], [68]. Previous studies have found that acetogenesis is favored over methanogenesis at low soil pH [69], low temperature [33] and in *Sphagnum*-dominated areas [70], all of which apply to our site. However, there is no metagenomic evidence that acetogenesis is more important than methanogenesis, and both hydrogen and actetate-utilizing clades of methanogens were found. *Methanoregula* is an acidiphilic methanogen [71] found extensively in these metagenomes (10% of all methanogenic sequences), possibly indicating a low pH-adapted methanogenic community. The H_2_ concentration at this site is relatively low [7], and this could give an advantage to hydrogenotrophic methanogens over acetogens in competition for H_2_ [72].

Metagenomic studies of complex microbial communities describe metabolic potential rather than identifying which processes occur at any given time. At the broadest subsystem level, the suite of functional categories present within metagenomes can be quite similar between two environments greatly differing in taxonomy or physicochemical properties [73]. This probably results from the great diversity of natural microbial communities in comparison to a relatively smaller set of ways they can make a living in the environment. However, metagenomes are especially useful in generating hypotheses to be tested with more targeted approaches. In this study we used metagenomic data to identify specific anaerobic processes whose rates should vary with depth in the soil profile, and we proposed that specific taxa and genes are involved in these processes. Future work should explicitly test such hypotheses from metagenomic studies. The currently available metagenomic libraries from Arctic soils have been based on relatively few individual soil cores; an understanding of the full spatial and functional heterogeneity of the Arctic microbiome is still developing.
